# Overcoming Immune Therapy Resistance in Cancer Through Innate Immune Reprogramming

**DOI:** 10.3390/ijms26199554

**Published:** 2025-09-30

**Authors:** Giada Mandracci, Nardine Soliman, Nadia El Khawanky

**Affiliations:** 1 Department of Medicine III, School of Medicine and Health, Technical University of Munich, 81675 Munich, Germany; giada.mandrax@gmail.com (G.M.); nardine.soliman@tum.de (N.S.); 2 TranslaTUM, Center for Translational Cancer Research, Technical University of Munich, 81675 Munich, Germany

**Keywords:** innate immunity, immune therapy resistance, cancer immunotherapy, tumor microenvironment, nucleic acid sensing, pattern recognition receptors, STING pathway, TLRs, RIG-I, MDA5, dendritic cells

## Abstract

Overcoming immune resistance remains the critical barrier to durable immunotherapy responses. Tumors with non-inflamed, “cold” microenvironments exclude cytotoxic lymphocytes and evade checkpoint blockade. Innate nucleic acid-sensing pathways—including TLRs, RIG-I-like RNA sensors, and the cGAS–STING DNA-sensing axis—can recondition this hostile landscape by licensing dendritic cells, restoring antigen presentation, and recruiting effector T and NK cells. In this review, we synthesize mechanistic insights into how these receptors function across tumor and immune compartments and evaluate recent translational advances spanning small-molecule and nucleic acid agonists, engineered delivery systems, and clinical trials. We highlight challenges that have limited clinical impact, including pathway silencing, systemic toxicity, and lack of predictive biomarkers, while emphasizing emerging solutions such as tumor-intrinsic targeting, CAR-T/NK engineering, and biomarker-guided patient selection. By integrating innate activation into rational combination regimens, innate immune reprogramming offers a blueprint to convert resistant disease into one susceptible to durable immune control.

## 1. Introduction

The advent of immunotherapy has transformed the landscape of cancer treatment, delivering unprecedented clinical benefits across various malignancies once considered untreatable. Immune checkpoint inhibitors (ICIs) directed against PD-1/PD-L1 and CTLA-4, alongside adoptive cellular therapies (ACT) such as chimeric antigen receptor (CAR)-T and CAR-natural killer (NK) cells, have established a new therapeutic paradigm. Yet, the promise of immunotherapy remains uneven. A substantial proportion of patients exhibit primary resistance, while others relapse following initial response, reflecting the pervasive challenge of immune escape. These limitations underscore the need for complementary strategies capable of broadening and deepening therapeutic efficacy across diverse cancers.

At the heart of this challenge lies the tumor microenvironment (TME)—a dynamic, complex, and frequently immunosuppressive ecosystem. The TME actively constrains immunotherapy efficacy through mechanisms such as impaired antigen presentation, exclusion and/or exhaustion of tumor-infiltrating immune cells, and the recruitment of regulatory or myeloid-derived suppressor cell (MDSC) populations. Notably, tumors often described as “cold”—manifesting immune-desert or immune-excluded phenotypes—exemplify environments that are refractory to immunotherapeutic engagement. Overcoming these entrenched barriers necessitates approaches that fundamentally reshape or reprogram the immune landscape at its foundation.

Innate immune sensing pathways provide a compelling rationale for such interventions. Pattern recognition receptors (PRRs) such as Toll-like receptors (TLRs), RIG-I-like receptors (RLRs), as well as the cyclic GMP-AMP synthase (cGAS)-stimulator of interferon genes (STING) pathway axis, serve as first-line detectors of aberrant nucleic acids. Their activation orchestrates robust type I interferon (IFN-I) and proinflammatory responses that enhance antigen presentation, recruit and activate effector cells, and recalibrate suppressive niches. In this way, innate nucleic acid sensing pathways can convert “cold” tumors into inflamed, immunologically “hot” states, thereby offering strategies to overcome resistance to existing therapies.

This review will examine how modulation of nucleic acid sensing pathways can be harnessed to fortify antitumor immunity. We highlight the mechanistic basis of their function in cancer, summarize preclinical and early clinical advances, and consider how these strategies can be integrated with established immunotherapeutic modalities. By focusing on innate immune reprogramming, we aim to illuminate a promising frontier in cancer immunotherapy that leverages endogenous immune circuitry to enable broader and more durable clinical responses.

## 2. The Tumor Immune Microenvironment: Composition and Role in Resistance

Immune cells within the TME operate within an intricate and finely tuned network of cooperation and conflict that collectively determines whether a tumor is eliminated, restrained, or allowed to progress. At one side of this spectrum, cytotoxic CD8^+^ T lymphocytes and NK cells form the core executioners of anti-tumor immunity [1]. They eliminate malignant cells through targeted cytolysis and by releasing inflammatory mediators that recruit and activate additional immune effector populations. On the opposing side, regulatory T cells (Tregs), MDSCs, and immunosuppressive macrophage subsets work in concert to attenuate cytolytic activity, disrupt antigen recognition, and reinforce tolerance to tumor antigens [2,3,4]. The balance between these counteracting forces—and their spatial organization within or around tumor nests—underpins the immune-inflamed (“hot”), immune-excluded, and immune-desert (“cold”) phenotypes that strongly predict therapeutic responsiveness [5].

CD8^+^ T cells are key actors in adaptive immunity and recognize tumor-associated antigens (TAAs) presented on major histocompatibility complex (MHC)-I molecules and induce apoptosis through perforin–granzyme release, FAS/FAS ligand (FASL) engagement, and interferon-gamma (IFN-γ) secretion [6,7]. Beyond their cytolytic function, IFN-γ amplifies immune responses by enhancing antigen presentation in surrounding tumor and stromal cells and by recruiting additional immune effector populations into the TME.

NK cells complement and sometimes precede T cell activity by rapidly detecting transformed or stressed cells with diminished MHC-I expression, initiating cytolysis without prior antigen sensitization [8,9,10]. Importantly, the actions of these two cell types are mutually reinforcing: T cell-derived cytokines augment NK cell activation, while NK cell-mediated tumor lysis releases antigens that dendritic cells (DCs) can capture, further fueling the T cell response [1].

DCs are pivotal in linking innate and adaptive immunity. They patrol the tumor site, capture antigens from dying tumor cells, and migrate to lymphoid tissues to prime naïve T cells. As the “teachers” of the immune system, dendritic cells are critical for shaping effective immunity. Impairments in their function—such as reduced co-stimulatory molecule expression or a tolerogenic shift driven by interleukin-10 (IL-10) and transforming growth factor-β (TGF-β)—can disrupt the broader immune network and weaken cytotoxic responses [11,12]. Similarly, tumor-associated macrophages (TAMs) embody the same duality: when polarized toward a pro-inflammatory M1-like state, they can present antigens and produce inflammatory cytokines that sustain T and NK cell activity. Conversely, when polarized toward an M2-like state, they secrete IL-10, TGF-β, and indoleamine 2,3-dioxygenase (IDO), depleting local nutrients such as tryptophan. This creates a biochemical environment that suppresses effector function and promotes regulatory populations [13,14,15].

These cellular interactions are further shaped by molecular and structural barriers that collectively establish immune resistance. Tumors frequently downregulate MHC-I expression or components of the antigen-processing machinery, including transporter associated with antigen processing (TAP) proteins, thereby evading recognition by CD8^+^ T cells [16]. Over time, selective immune pressure can drive the loss of immunogenic neoantigens—a process akin to “antigenic drift”—allowing resistant subclones to persist [17]. The cytokine milieu adds another layer of control: IL-10 and TGF-β impair DC activation, dampen NK cell cytotoxicity, and promote Treg expansion, while IL-6 and IL-1β reinforce chronic inflammation that paradoxically favors tumor progression through epithelial–mesenchymal transition (EMT) [12].

Angiogenesis also intersects with immune regulation. Vascular endothelial growth factor (VEGF) promotes a disorganized, leaky vascular network that not only facilitates metastasis but also limits lymphocyte entry into the tumor parenchyma [18,19]. VEGF additionally contributes to immunosuppression by inhibiting DC maturation and recruiting suppressive myeloid populations.

Even when effector cells successfully infiltrate tumors, chronic antigen exposure, hypoxia, and nutrient depletion drive T cell exhaustion, marked by the sustained expression of inhibitory receptors such as PD-1, CTLA-4, TIM-3, and LAG-3 [20,21,22]. Exhausted T cells exhibit diminished responsiveness to stimulatory cues, enabling suppressive populations including Tregs, MDSCs, and M2-like TAMs to dominate the immune landscape [23].

Critically, these resistance mechanisms rarely act in isolation. Rather, they form an interconnected network in which defective antigen presentation, immunosuppressive cytokines, pro-angiogenic factors, metabolic constraints, and cellular suppressors reinforce one another to sustain an environment hostile to antitumor immunity. Consequently, therapeutic strategies aiming to overcome resistance must target multiple nodes within this network—simultaneously restoring antigen visibility, blocking inhibitory pathways, normalizing vasculature, and reprogramming suppressive immune populations into pro-inflammatory phenotypes.

## 3. Innate Nucleic Acid Sensing Pathways in Tumor Immunity

**Figure 1:**
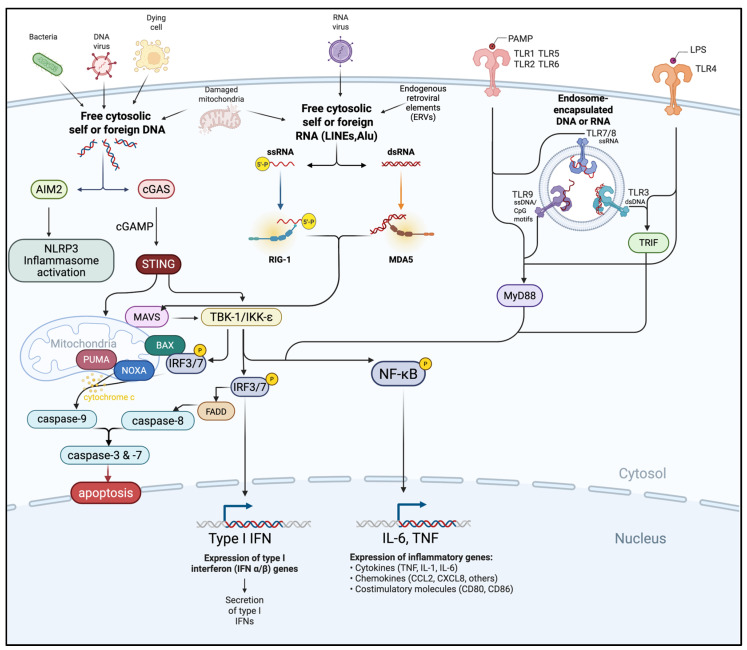
Crosstalk of cytosolic and endosomal innate immune sensing pathways. Foreign or aberrantly located self-DNA and RNA derived from pathogens, dying cells, damaged mitochondria, or endogenous retroviral elements (ERVs) are detected by cytosolic (AIM2, cGAS, RIG-I, MDA5, TLR1, TLR2, TLR5/6, TLR4) or endosomal sensors (TLR3, TLR7/8, TLR9). These pathways converge on adaptor proteins (STING, MAVS, MyD88, TRIF) and downstream kinases (TBK1/IKKε) to activate transcription factors (IRF3/7, NF-κB), pro-apoptotic mediators, and caspases. The resulting responses include IFN-I and pro-inflammatory cytokine production, inflammasome activation, and induction of apoptosis. Figure created using Biorender.com.

The innate immune system deploys a network of nucleic acid sensors (Figure 1) that act as the first line of defense against pathogens and cellular abnormalities, including those arising in cancer. These receptors detect molecular patterns associated with viral or bacterial nucleic acids and, in the tumor context, recognize danger signals derived from genomic instability, DNA damage, or aberrant RNA transcription [24]. Despite their distinct ligand specificities and subcellular localizations, they feed into a shared downstream signaling architecture mediated by interferon regulatory factor (IRF)3/7 and nuclear factor-kappa B (NF-κB), triggering robust production of IFN-I and proinflammatory cytokines [25,26]. Beyond IFN-I, this coordinated signaling orchestrates a multifaceted immune response, promoting DC maturation, enhancing antigen presentation, facilitating cross-priming of CD8^+^ T cells, and activating NK cells, which then release cytotoxic granules and produce IFN-γ.

Importantly, many of these pathways also induce immunogenic cell death (ICD), a regulated process of apoptosis or necroptosis that transforms dying tumor cells into pro-inflammatory beacons. ICD is characterized by the release or exposure of danger-associated molecular patterns (DAMPs), including calreticulin, adenosine triphosphate (ATP), and high-mobility group box 1 (HMGB1), which synergistically recruit and activate DCs. Calreticulin functions as an “eat-me” signal, ATP acts as a chemoattractant and activates the NLR family pyrin domain containing 3 (NLRP3) inflammasome, and HMGB1 engages TLR4 and other receptors to enhance antigen processing. This distribution enables multi-tiered immune surveillance in which both tumor and immune cells can act as sources and targets of danger signals [26,27].

While these pathways have historically been studied in antiviral immunity, these sensors are increasingly being recognized as central modulators of the TME. Collectively, nucleic acid sensing pathways—including TLRs, RLRs, and the cGAS–STING pathway—coordinate immune activation that extends well beyond IFN-I production, reshaping the tumor-immune microenvironment and tipping the balance toward a more immunologically “hot” and therapy-responsive cancer.

### 3.1. Toll-like Receptors (TLRs)

TLRs are evolutionarily conserved endosomal sensors of microbial and endogenous nucleic acids, serving as sentinels of both infection and cellular distress. In cancer, TLR signaling can have dichotomous effects; it promotes antitumor immunity by driving DC maturation, antigen presentation, and effector cell recruitment, while in other cases, it sustains tumor-promoting inflammation, angiogenesis, immune evasion, and EMT. The overall effect depends on the cancer type, tumor stage, and the nature of nucleic acid ligands present within the TME.

Among the best-characterized nucleic acid sensors are the endosomal TLRs—TLR3, TLR7, TLR8, and TLR9—that recognize both microbial nucleic acids and endogenous tumor-derived ligands. DCs, particularly conventional type 1 DCs (cDC1s), are enriched in TLR3 and play a specialized role in cross-presenting tumor antigens to CD8^+^ T cells. Plasmacytoid DCs (pDCs) express high levels of TLR7 and TLR9, enabling rapid IFN-I release, thus priming cytotoxic lymphocytes and activating NK cells. Macrophages, especially M1-like phenotypes, engage TLR pathways to amplify inflammation; in contrast, M2-like macrophages may suppress these signals through IL-10 secretion and metabolic modulation. NK cells themselves, although less sensor-rich, respond robustly to TLR-induced cytokines and chemokines derived from DCs and tumor cells, forming reciprocal activation loops that enhance cytotoxicity [28]. Importantly, TLRs are also expressed in tumor cells, where activation can directly modulate survival, proliferation, therapy resistance, and apoptosis, reflecting the duality of tumor-intrinsic versus immune-extrinsic TLR effects.

#### 3.1.1. TLR3

TLR3 recognizes double-stranded RNA (dsRNA) and is expressed in specialized DC subsets such as CD103^+^ DCs in mice and their human homologs [29]. In cancer, dsRNA ligands arise not only from viruses but also from dysregulated transcription of endogenous retroviruses (ERVs), long interspersed nuclear elements (LINEs), and Alu repeats, as well as bidirectional transcription of defective RNA processing [30,31,32]. Normally, loss of ADAR1-mediated editing, as seen in hepatocellular carcinoma and T cell acute lymphoblastic leukemia (T-ALL), results in the accumulation of dsRNA and subsequent activation of TLR3 [33,34].

Clinically, adjuvant dsRNA therapy (poly A:U) reduced metastatic relapse in breast cancer, specifically in patients with TLR3-positive tumors [35]. Beyond immune cells, TLR3 is expressed in various tumor types, where its engagement can have direct cytotoxic effects. For instance, Poly I:C stimulation induces apoptosis in prostate LNCaP cells through an IRF3-dependent mechanism and suppresses tumor growth in vivo. Similar effects have been observed in melanoma cell lines, especially following IFN-I priming, and poly I:C also reduces clonogenic potential in hepatoma cells. Conversely, in some epithelial cancers, TLR3 activation has been linked to pro-survival NF-κB signaling and increased resistance to chemotherapy [36].

#### 3.1.2. TLR7 and TLR8

TLR7 and TLR8 detect single-stranded RNA (ssRNA) and are primarily expressed in pDCs (TLR7), B cells (TLR7), and myeloid cells (TLR8). Their activation promotes IFN-I secretion and inflammatory signaling that enhance DC maturation and T cell priming [37,38]. In tumors, ssRNA ligands may originate from several sources: (i) aberrant transcription of retroelements such as LINEs, short interspersed nuclear elements (SINEs), and ERVs, which are frequently derepressed in cancer due to epigenetic instability; (ii) RNA released by apoptotic or necrotic tumor cells; and (iii) dysregulated noncoding RNAs such as microRNAs and long noncoding RNAs [39,40,41]. For instance, tumor-derived exosomes carrying miR-21 and miR-29a activate TLR8 in human macrophages (and TLR7 in murine models), leading to NF-κB activation and protumorigenic cytokine release in lung cancer [42]. Conversely, therapeutic siRNAs targeting viral oncogenes (E6/E7) in HPV-associate cancers can silence these oncogenes while simultaneously stimulating TLR7/8, thereby boosting innate immune activation [43]. Tumor cells themselves may also express TLR7/8, and their activation has been associated with increased proliferation, survival, and chemoresistance in pancreatic, lung, and breast cancers [44,45]. On the other hand, in certain leukemias, TLR7 stimulation can trigger apoptosis, illustrating how the tumor-intrinsic consequences of TLR7/8 signaling are context-dependent [46,47].

#### 3.1.3. TLR9

TLR9 detects unmethylated CpG motifs, recognizing not only microbial DNA but also tumor-derived mitochondrial and nuclear DNA fragments released during genomic instability or cell death [48,49]. Importantly, the abundance of immunostimulatory CpG motifs in cancer-derived DNA is partly explained by epigenetic reprogramming during tumorigenesis [50]. While normal mammalian DNA is generally CpG-methylated and thus less stimulatory, cancer cells frequently exhibit global DNA hypomethylation, particularly within repetitive sequences and intergenic regions. Global DNA hypomethylation, a hallmark of tumorigenesis, arises from dysregulation of DNA methyltransferases (DNMTs), aberrant activity of ten-eleven translocation (TET) family demethylases, and replication-associated loss of methylation fidelity in rapidly dividing cells [50]. This process produces abundant unmethylated CpG-rich DNA fragments which, when released into the TME through apoptosis, necrosis, or extracellular vesicles (EVs), act as endogenous DAMPs that activate TLR9 and amplify innate immune signaling [51,52]. Besides pDCs and B cells, TLR9 expression is prominent in certain hematologic malignancies such as chronic lymphocytic leukemia (CLL), where it may paradoxically skew signaling toward tumor-supportive NF-κB activation rather than robust IFN responses [53]. Equally, tumor-intrinsic TLR9 signaling has been implicated in diverse outcomes where CpG engagement has shown to drive tumor growth, invasion, and chemoresistance via NF-κB and STAT3 activation in breast and glioma models [54,55]. Conversely, TLR9 stimulation can enhance apoptosis and sensitization of malignant cells to DNA-damaging therapies in melanoma [56].

TLR engagement upregulates co-stimulatory molecules (CD80, CD86, CD40) as well as MHC class I/II on DCs, delivering potent activation signals to naïve T cells and skewing immunity toward T helper 1 (Th1)-mediated cytotoxicity through pro-inflammatory cytokines, including IL-2, IL-6, and TNF-α [57,58,59]. This cytokine milieu licenses NK cells with IL-12 and IL-15, enhancing their cytolytic activity and IFN-γ secretion, further augmenting antigen presentation and lymphocyte tumor infiltration. Chemokines such as CXCL9, CXCL10, and CCL5 produced by both tumor cells and immune cells reinforce recruitment of effector CD8^+^ T cells and NK cells into the tumor [60]. Additionally, TLR signaling also mitigates protumorigenic aspects of the TME by limiting abnormal angiogenesis and reprogramming immunosuppressive cellular subsets like Tregs and MDSCs [57,58].

TLR activation can also invoke ICD, releasing nucleic acids that fuel cytosolic sensors like RLRs and cGAS-STING, thereby amplifying immune activation cycles within the tumor [61,62]. However, tumors may also evade such responses by suppressing sensor pathways through epigenetic silencing or the expression of negative regulators like three primer repair exonuclease (TREX1) or ectonucleotide pyrophosphatase/phosphodiesterase 1 (ENPP1). In certain contexts, these responses can even be hijacked to support tumor-promoting inflammation and immune evasion [63,64,65].

Despite these mechanisms of evasion and context-dependent protumorigenic effects, the potent immunostimulatory capacity of TLRs has spurred efforts to therapeutically harness their activation. TLR agonists are currently being tested as adjuvants or in combination with checkpoint blockade, vaccines, or adoptive cellular therapies. In preclinical models of breast, melanoma, and lung cancer, TLR agonists have been shown to enhance ICD, promote NK cell infiltration, and improve T cell priming, thereby overcoming resistance to PD-1/PD-L1 or CTLA-4 blockade. Early-phase clinical trials of CpG oligodeoxynucleotides (TLR9 agonists) or imiquimod (TLR7 agonist) demonstrate localized enhancement of immune infiltration and increases in systemic antitumor responses when used in combination with checkpoint inhibitors. The translational promise lies in harnessing these effects while avoiding the deleterious consequences of chronic or systemic TLR activation, which can promote immune exhaustion or tumor-supportive inflammation [66,67,68,69].

### 3.2. Cytoplasmic RNA Sensors: RIG-I and MDA5

RLRs are cytoplasmic sensors expressed both in tumor and immune cells. They specialize in detecting viral-like RNA molecules and act as an intracellular alarm system against infection and cellular transformation [70]. In cancer, activation of these sensors mimics an antiviral state, triggering IFN-I production, promoting antigen presentation, and directly inducing tumor cell death. Retinoic acid-inducible gene I (RIG-I) and melanoma differentiation-associated gene 5 (MDA5) are the two best-characterized cytosolic RLRs that complement endosomal pathogen recognition by detecting viral-like RNA within the cytosol. RIG-I preferentially recognizes short dsRNA or ssRNA with 5′ triphosphate or diphosphate ends, whereas MDA5 detects long dsRNA species. Upon ligand binding, both undergo conformational changes that enable interaction with the mitochondrial antiviral-signaling protein (MAVS) [71].

A distinctive feature of RLR signaling is its activation by endogenous RNA species that accumulate in cancer cells following genotoxic stress, DNA damage, or metabolic alterations [72,73,74]. For example, ionizing radiation or chemotherapy induce the accumulation of small endogenous non-coding RNAs with double-stranded structures that selectively activate RIG-I [75,76]. Moreover, laboratory of genetics and physiology 2 (LGP2) acts as a specific regulator of RLR-driven responses modulating radiosensitivity [77]. RIG-I employs multilayered RNA recognition and proofreading mechanisms to distinguish viral or aberrant RNAs from host RNAs. Its ATPase and helicase activities fine-tune RNA binding and downstream signaling, preventing harmful autoinflammation [72,78]. Upon ligand recognition and oligomerization, the caspase activation and recruitment domain (CARD) domains of RLRs interact specifically with MAVS on the mitochondrial membrane. This interaction activates IRF3/7 and NF-κB, producing a strong IFN-I response.

In parallel, RLR signaling can trigger a pro-apoptotic cascade independent of IFN, characterized by upregulation of Bcl-2 homology 3 (BH3)-only proteins such as Noxa and Puma in multiple cancers including melanoma, pancreatic, ovarian, breast, and hematologic malignancies [79,80,81,82]. This dual pathway culminates in mitochondrial apoptosis specifically in tumor cells, which frequently lack the anti-apoptotic safeguard mediated by proteins like B-cell lymphoma-extra large (Bcl-xL) that protect normal cells. As a result, tumor cells are more sensitive to RIG-I- and MDA5-induced apoptosis than non-malignant cells, making this pathway exploitable for selective tumor targeting [79,80,83]. The cell death pathways driven by RIG-I and MDA5 are not merely byproducts of inflammation but represent distinct mechanisms of tumor suppression that differ from TLR-induced inflammatory cell death.

Beyond direct tumor cell death, RLR activation also boosts NK cell cytotoxicity through TRAIL-mediated mechanisms, promotes immunogenic cell death with DAMP exposure, and enhances antigen cross-presentation by DCs [84,85]. In addition, tumor cells stimulated via RLR pathways secrete immunostimulatory EVs. These EVs are enriched in RNA and protein cargo that can activate pattern recognition receptors on surrounding immune cells, spreading immune activation beyond the initially targeted tumor cells [86,87,88]. Through this mechanism, individual tumor cells undergoing RLR response can ignite broader immune crosstalk across the TME. These specialized RNA recognition and regulatory mechanisms allow the RLR pathway to balance strong anti-tumor immunity while limiting the risk of systemic autoimmunity.

### 3.3. Cytosolic DNA Sensing: The cGAS–STING Pathway

The cGAS–STING pathway is a central cytosolic DNA sensing mechanism that detects double-stranded DNA (dsDNA) misplaced in the cytoplasm. This cellular event is a hallmark of viral infection but also frequently occurs during tumorigenesis [89,90]. In cancer, cytosolic dsDNA often arises as a byproduct of genomic instability, replication stress, oxidative damage, and impairments in DNA repair pathways such as homologous recombination deficiencies (as seen in BRCA-mutant tumors). Errors in chromosome segregation generate micronuclei that can rupture, releasing DNA into the cytosol. Meanwhile, mitochondria under metabolic stress or defective mitophagy release mitochondrial DNA [91,92]. These processes intimately link the hallmark drivers of tumor growth—chromosomal instability, replication stress, defective repair, and metabolic dysfunction—to innate immune activation through cGAS.

Upon binding cytosolic dsDNA, cGAS is activated to catalyze the synthesis of cyclic GMP-AMP (cGAMP), a cyclic dinucleotide second messenger. cGAMP binds to the adapter protein STING, located on the endoplasmic reticulum (ER) membrane, triggering the translocation of STING from the ER to the ER–Golgi intermediate compartment and subsequently to the Golgi apparatus. This translocation is crucial for recruiting and activating the canonical downstream signaling pathway [93]. In addition to activating IFN-I responses, STING-driven signaling induces the production of chemokines such as CXCL10, which can convert immune-desert (“cold”) tumors into immune-inflamed lesions, thereby enhancing their responsiveness to existing therapies [94,95,96].

Besides the canonical tank-binding kinase 1 (TBK-1) mediated IRF3 and NF-κB signaling, STING can also engage non-canonical NF-κB pathways, thereby initiating inflammatory signaling cascades with broad implications. Notably, activation of non-canonical NF-κB has been implicated in driving EMT, a process that confers enhanced motility and invasiveness on cancer cells, ultimately facilitating metastatic spread and resistance to cell death [97]. STING activation also exerts profound effects on organelle homeostasis. Its trafficking to the Golgi and ER induces ER stress, which in turn promotes autophagy—a catabolic program that can enable tumor cells to withstand metabolic and therapeutic stressors [98]. However, this effect is context-dependent: while moderate autophagy promotes survival, persistent ER stress can culminate in apoptotic or necroptotic cell death. Thus, the cGAS–STING axis functions as a rheostat, balancing pro-survival and pro-death outcomes within the TME.

Beyond NF-κB and autophagy, STING signaling intersects with inflammasome biology. The activation of the NLRP3 inflammasome downstream of cGAS–STING triggers the release of IL-1β and other pro-inflammatory mediators [99], thereby potentially shaping a TME that can either support antitumor immunity or, paradoxically, promote tumor growth through chronic inflammation. With tumor persistence, chronic STING activity can also enforce a senescent state characterized by permanent growth arrest. Senescent cancer cells secrete a distinct profile of cytokines, chemokines, and immune modulators collectively known as the senescence-associated secretory phenotype (SASP) [100,101]. Components of the SASP, including PD-L1 and TGF-β, remodel the tumor milieu toward immunosuppression and therapeutic resistance.

At the molecular level, cGAS–STING signaling modulates both genomic stability and metabolic programming. By influencing DNA repair pathways, STING activity alters the mutational landscape of tumors, thereby shaping their evolutionary trajectory. In parallel, STING activation reconfigures cellular metabolism to favor survival under stress, reinforcing its role as a central regulator of tumor adaptation [102,103]. Importantly, the cGAS–STING pathway is not restricted to malignant cells but is also active in diverse populations within the TME, including immune cells and cancer-associated fibroblasts (CAFs) [104,105,106]. Through these compartments, STING signaling contributes both to immunostimulatory and immunosuppressive programs, thereby influencing tumor progression and modulating therapeutic response. Conversely, certain cancers actively evade recognition. For example, acute myeloid leukemia (AML) blasts downregulate cytosolic DNA and RNA sensors to avoid activation of type I interferon–producing pDCs [107,108]. Moreover, the bone marrow microenvironment—through hypoxia, altered metabolite levels, and stromal interactions—further suppresses nucleic acid sensing pathways, reinforcing immune evasion [109].

A particularly striking feature of the cGAS–STING axis is its ability to propagate signaling beyond the cell of origin. Tumor cells can export cGAMP through gap junctions or EVs, thereby activating STING in neighboring tumor and stromal cells. In brain metastases, cancer-derived cGAMP activates STING in astrocytes, driving inflammation that fosters metastasis and chemotherapy resistance—a signaling mode not seen with other nucleic acid sensors [110,111].

Together, TLRs, RLRs, and cGAS–STING form a tightly integrated network that drives immunogenic tumor cell death, reshapes the tumor-immune microenvironment, and enhances adaptive immunity, providing critical mechanistic foundations for combination cancer immunotherapies.

## 4. Leveraging Innate Immunity to Overcome Resistance and Enhance Immunotherapy

### 4.1. Mechanisms and Preclinical Advances in Activating Innate Immune Cells Within the TME

**Figure 2:**
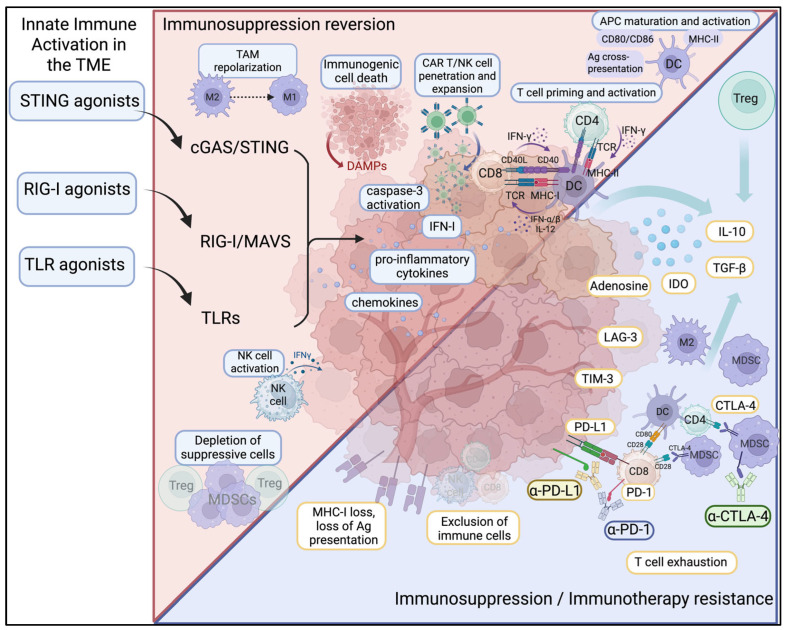
Innate immune activation reverses immunosuppression and immunotherapy resistance in the tumor microenvironment. Solid tumors employ immunosuppressive mechanisms such as loss of MHC-I, downregulation of the antigen-presentation machinery, infiltration of MDSCs and Tregs, and upregulation of inhibitory checkpoints (PD-1/PD-L1, CTLA-4, LAG-3) and immunosuppressive cytokines (IL-10, TGF-β), which drive resistance toward immune checkpoint blockade and adaptive CAR T/NK cell therapies. Agonists of STING, RIG-I, and TLR pathways activate innate immune signaling within the TME, leading to the production of IFN-I, pro-inflammatory cytokines, and chemokines, as well as inducing immunogenic cell death. These mechanisms collectively reprogram the cold TME into a hot tumor milieu, promoting maturation and activation of DCs, priming of antigen-specific T cells, NK cell activation, TAM repolarization, and depletion of suppressive cells, ultimately enhancing anti-tumor immunity and reversing therapy resistance. Figures created using BioRender.com.

Activation of innate immune pathways using agonists targeting STING, TLRs, and RIG-I has demonstrated significant preclinical promise for reprogramming the TME from immunosuppressive to immunostimulatory (Figure 2). STING and TLR activation polarize TAMs away from the immunosuppressive M2 phenotype toward pro-inflammatory M1-like states, enhancing antigen presentation and cytokine release. This has been observed in breast cancer models where STING agonists reduce M2 markers (e.g., Retnla, Folr2) and increase M1-associated chemokines such as CXCL9, CXCL10 and NOS2 [112]. In such models, STING engagement also re-sensitizes BRCA1-deficient tumors to Poly (ADP-ribose) polymerase (PARP) inhibitors, underscoring the cooperation between innate immunity and the DNA damage response [113]. Modulation of Fcγ receptor activatory:inhibitory ratios on TAMs by STING agonists has also been shown to support enhanced phagocytosis of tumor cells in overcoming resistance to antibody-based therapies in vivo [114].

Similarly, class C TLR9 agonists promoted local immune activation by reducing suppressive MDSCs and increasing M1/M2 macrophage ratios in liver metastases [115,116]. Meanwhile, STING-driven type I interferons amplified NK cell cytotoxicity and promoted TCF1^+^ memory-like NK subsets that are critical for effective tumor clearance, as seen in glioblastoma models [117]. Novel delivery systems, such as virus-like particles carrying cGAMP or ER-targeted STING-antigen conjugates (e.g., SABER), amplify CD8^+^ T cell responses through the activation of cDC1 and cDC2 subsets [118,119].

RIG-I agonists, due to their ubiquitous expression in the TME, stimulate both tumor cells and resident immune cells. In murine melanoma, both local and systemic activation of RIG-I promotes the enrichment of activated NK cells, CD11b^+^ myeloid cells, and pDCs, alongside a robust cytokine release [120,121]. Furthermore, RIG-I agonists induce immunogenic cell death, which facilitates the release of pro-inflammatory cytokines and chemokines, triggering DC activation and maturation that collectively support antigen-specific CD8^+^ T cell responses in human ovarian and murine pancreatic models [122].

An important innate-adaptive immune axis involves natural killer group 2D (NKG2D) receptors and their stress-induced ligands, MHC class I chain-related proteins A and B (MICA/B) and UL16-binding proteins (ULBPs). These ligands are upregulated on stressed or transformed tumor cells, serving as “distress signals” that activate NK cells and cytotoxic T lymphocytes (CTLs). However, tumor cells evade immune surveillance by shedding soluble forms of MICA/B and ULBPs, which downregulate NKG2D receptor expression on effector cells and impair their cytotoxic capacity [123,124,125]. In addition to ligand shedding, tumors secrete soluble factors such as lactate dehydrogenase 5 (LDH5), which drive systemic expression of NKG2D ligands (notably MICB and ULBP-1) on host myeloid cells. As shown by Crane et al., [126] this mechanism extends beyond glioblastoma, since NKG2D ligand-positive monocytes have also been detected in breast, prostate, and hepatocellular carcinomas. Prolonged exposure of NK cells to these ligand-bearing myeloid cells leads to functional inactivation of the NKG2D pathway, revealing an underappreciated route of immune evasion: tumors can subvert NK cell surveillance not only by shedding ligands but also by inducing them on host immune cells.

Interestingly, certain NKG2D ligands are regulated by tumor-intrinsic DNA damage response (DDR) signaling. For example, expression of retinoic acid early transcript 1 (RAE1) in lymphoma is controlled by STING-dependent DNA sensing. Lam et al. demonstrated that genomic instability activates STING, which signals through TBK1 and IRF3 to upregulate RAE1, thereby enhancing NK cell-mediated immunosurveillance. In murine lymphoma, IRF3 loss reduced RAE1 expression and was associated with diminished survival, underscoring the pivotal role of STING in promoting innate immune recognition via NKG2D ligands [127]. Additionally, therapeutic interventions inhibiting ligand shedding or neutralizing soluble ligands have shown to restore NK function and augment antibody-dependent cellular cytotoxicity (ADCC) in preclinical models [128,129].

Recent advances also include combination strategies expanding innate immune activation. Oncolytic viruses (OVs) naturally induce activation of RIG-I, MDA5, and various TLRs while releasing tumor antigens that enhance cross-presentation and adaptive immune priming. These OVs have shown synergistic effects when combined with innate agonists and ICIs. Moreover, targeting inflammasomes and nucleotide-binding oligomerization domain-like receptors (NLRs), especially NOD2 and AIM2, potentiates antitumor responses by increasing IL-1β and IL-18 secretion, although these approaches remain preclinical for the most part [130,131,132,133,134]. C-type lectin receptor (CLR) agonists, such as β-glucans targeting Dectin-1, prime DCs and macrophages to mount pro-inflammatory responses that enhance ICI efficacy [135,136]. In addition, nanoparticle (NP)-based delivery of multi-agonist formulations co-administering STING, TLR7/8, or RIG-I ligands alongside chemotherapy, radiotherapy or ICI have shown improved immune activation and tumor control compared to single agents [137,138,139]. In one such study, co-administration of an NP-based STING agonist with anti-PD-1 antibodies demonstrated marked tumor regression, even of checkpoint-resistant tumors, with significantly reduced systemic toxicity relative to free agonists [140]. Furthermore, integrating innate agonists with metabolic modulators, including adenosine/A2A receptor antagonists and IDO inhibitors, further attenuates immunosuppressive networks within the TME [141,142].

Clinical translation of these preclinical insights has accelerated with a growing number of oncology clinical trials exploring innate immune agonists, including STING and TLR pathway agonists, either as monotherapies or combined with ICIs, radiotherapy, vaccines, or cellular therapies. These trials span diverse tumor types, such as lymphomas, hepatocellular carcinoma, melanoma, pancreatic cancer, breast cancer, and sarcoma, employing a variety of delivery routes including intratumoral (i.t.), intravenous (i.v.), subcutaneous (s.c.), and topical applications. Predominantly, these studies are early-phase first-in-human trials focusing on safety, tolerability, and determination of recommended Phase II or maximum tolerated doses. Clinical strategies combining ICIs, CAR-T cells, and tumor vaccines in solid tumors are summarized in Table 1 (end of Section 4.2).

### 4.2. Innate Immune Agonists Potentiate Checkpoint Inhibition but Clinical Translation Highlights Limitations

#### 4.2.1. Innate Immune Agonists in Preclinical Models

ICI therapies targeting PD-1, CTLA-4, and LAG-3, have revolutionized cancer therapy by reinvigorating exhausted T cells. However, their efficacy remains primarily limited to immunologically “hot” tumors characterized by high T cell infiltration and antigen presentation. Numerous tumors, instead, present as immunologically “cold”, exhibiting poor antigenicity, limited immune infiltration, and an immunosuppressive TME [143,144]. Overcoming this barrier remains a major objective in immuno-oncology.

Preclinical models have consistently shown that innate pathway activation via STING, TLR, and RIG-I agonists promotes upregulation of MHC class I and co-stimulatory molecules on tumor and immune cells, driving type I interferon and chemokine production, DC maturation, and robust CD8^+^ T cell priming and infiltration. STING agonists—including cyclic dinucleotides (CDNs) and non-nucleotide small molecules—are among the most intensely investigated innate immune activators in oncology. In murine models, CDNs induce profound CD8^+^ T cell infiltration, durable antigen-specific memory, and regression of both injected and distant tumors. Yet this potency has not been recapitulated in humans. A cautionary precedent was DMXAA, whose activity was restricted to murine STING and ultimately proved non-translatable. Synthetic analogues such as ML RR-S2 CDA, which broadly activate human polymorphic STING variants, validated the feasibility of cross-species targeting and confirmed robust preclinical efficacy [94]. However, these benefits have consistently required i.t. administration, raising serious doubts about scalability to real-world clinical practice where most lesions are inaccessible. Other studies demonstrated that STING agonism in hepatocellular carcinoma (HCC) enhanced IFN production from Kupffer cells alongside increased MHC-I expression, facilitating intrahepatic CD8^+^ T cell recruitment [145].

Equally, TLR2 agonism (Pam3CSK4) suppressed MDSC accumulation and promoted CD8^+^ T cell activation in HCC and brain tumors while TLR7/8 agonists (R848) reduced Tregs and enhanced effector T cell recruitment in pancreatic ductal adenocarcinoma (PDAC) and colon carcinoma models [146,147,148]. Administration of TLR9 i.t. has demonstrated synergistic memory CD8^+^ T cell (CD127^high^KLRG1^low^) reprogramming when combined with PD-1 blockade, in contrast to the more terminally differentiated phenotype CD127^low^KLRG1^high^ when administered alone [149].

Among these innate immune sensors, RIG-I has emerged as a particularly promising target. Preclinical studies suggest that RIG-I activation is more effective at converting immunologically “cold” tumors into inflamed phenotypes than at directly driving regression of established lesions, indicating a primary role in sensitization rather than direct cytotoxicity. In poorly immunogenic breast cancers, RIG-I signaling not only triggered immunogenic tumor cell death and cytokine modulation but also promoted leukocyte infiltration, a predictive marker of response to ICI. In non-immunogenic melanoma models, i.t. delivery of RIG-I agonists remodeled the TME, inducing regression of treated tumors while generating abscopal effects in distant, untreated lesions, accompanied by durable immune memory [150]. Moreover, stimulation of tumor-intrinsic RIG-I in human melanoma restored ICI sensitivity by enhancing CD8^+^ T cell recognition through *de novo* induction of antigen-presentation machinery and increased expression of ICAM-1 [151]. Mechanistically, targeting RIG-I in solid tumors has been shown to be indispensable for synergistic efficacy with ICI. This occurs through the promotion of tumor-intrinsic apoptosis, enhanced antigen cross-presentation by cDC1s, and expansion of antigen-primed CD8^+^ T cells [152]. Nonetheless, resistance mechanisms such as ADAR1-mediated RNA editing can blunt RIG-I signaling, representing a potential barrier to therapeutic efficacy [153].

#### 4.2.2. Clinical Evaluation of Innate Immune Agonists

Nonetheless, clinical trials using innate agonists have presented mixed outcomes reflecting significant translational hurdles. The prototypical CDN agonists, ADU-S100 and MK-1454, combined with anti PD-1 antibodies, demonstrated clear on-target immune activation and cytokine induction when administered i.t. in patients with solid tumors and lymphomas. However, clinical responses were variable (ORR ~10% for ADU-S100 and 50% complete or partial response for MK-1454), likely reflecting the enrollment of patients with late-stage disease and/or prior relapse following PD-1 blockade (Table 1). Whether these modest regressions were driven by direct tumor cell apoptosis or secondary immune priming remains unresolved, underscoring a mechanistic gap that complicates further clinical development [154,155].

Attempts to broaden feasibility with systemic STING agonists have so far failed to deliver. BMS-986301 was discontinued for lack of efficacy (NCT03956680), SB 11,285 has yet to report clinical outcomes despite advancing through early trials [156], and MK-2118 failed to achieve biologically active systemic exposure even at supratherapeutic s.c. doses [157]. Collectively, these findings highlight the ongoing uncertainty regarding the optimal route of delivery required to achieve therapeutically meaningful STING activation in solid tumors.

Pharmacodynamic signals observed in human studies further complicate interpretation. Elevated systemic levels of IFN-γ, CXCL10, and IL-6 consistently follow local STING activation, yet these biomarkers do not correlate with tumor regression, suggesting either rapid immune adaptation, feedback suppression, or even paradoxical protumorigenic effects of chronic STING signaling [157]. Preclinical evidence adds further complexity. In B cell malignancies such as leukemia, lymphoma and multiple myeloma, STING activation induces direct apoptosis in malignant B cells but with equal potency in normal B cells, raising concerns about an unacceptably narrow therapeutic window [114,158]. While STING agonism synergizes with antibody immunotherapy in B-cell lymphomas, its lack of selectivity poses translational risk.

At the mechanistic level, the cellular compartments mediating therapeutic benefit remain uncertain. While myeloid and dendritic cells are often assumed to be the critical STING-responsive populations, definitive evidence is lacking, and the contribution of tumor-intrinsic STING signaling is unclear. This ambiguity is compounded by frequent STING pathway silencing in solid tumors, often due to epigenetic repression, which may preclude tumor-intrinsic activity altogether and restrict efficacy to stromal or myeloid compartments [159,160,161,162]. Such context dependence likely contributes to the variability in clinical outcomes and underscores the need for biomarker-driven patient selection—an area still underdeveloped in ongoing trials.

Clinical evaluation of TLR agonists (Table 1)—whether as monotherapies or in combination with checkpoint inhibition—has produced highly variable outcomes, reflecting both biological and translational challenges. Key limitations include heterogeneous TLR expression across tumors and immune subsets, as well as the distinct subcellular localization of different TLRs (endosomal versus surface), which affects ligand accessibility and downstream signaling. This variability complicates predictions of responsiveness and likely contributes to inconsistent results. I.t. injection remains the predominant delivery route, but its practicality is limited to accessible lesions. Systemic approaches—including intravenous BDB001 (TLR7/8 agonist), antibody–TLR conjugates such as NJH395, and orally available agents like APR003 and SHR2150—remain exploratory, with limited efficacy data [156,163,164,165].

Among i.t. agents, CMP-001 (a TLR9 agonist) in combination with pembrolizumab has shown activity in advanced melanoma, particularly in “cold” tumors with minimal baseline immune infiltration characterized by low CD8^+^ T cell infiltration, minimal PD-L1 expression, and reduced IFNγ-related gene expression [166]. This suggests that TLR9 agonism can recondition a non-inflamed TME and restore responsiveness to checkpoint inhibition. However, responses have been inconsistent and frequently partial, raising questions about whether the observed effects are due to direct reprogramming of myeloid cells or secondary recruitment of effector T cells. By contrast, trials with LHC165 (a TLR7 agonist) in combination with spartalizumab highlight the opposite trend: clinical benefit appeared enriched in tumors with an already active immune milieu, where baseline IFN signatures predicted responsiveness [167]. These divergent outcomes emphasize the lack of a unifying mechanistic model for how different TLR agonists act within the TME and underscore the risk of treating all TLR agonists as functionally interchangeable.

RIG-I agonists have entered early-phase clinical testing but face similar translational barriers. In the first-in-human MK-4621 trial (Table 1), interferon-stimulated gene (ISG) induction confirmed target engagement, yet no objective responses were observed as monotherapy, and combination with pembrolizumab yielded only a modest benefit (ORR ~10%) [168]. A similar pattern was seen with CV8102, where immune activation was measurable but objective response rates were modest (3/33 monotherapy, ~17% in PD-1 refractory melanoma). These data highlight a recurrent challenge: RIG-I agonists produce robust pharmacodynamic “footprints” but weak clinical outcomes, raising the question of whether the readouts currently used (e.g., ISG induction, cytokine release) serve as appropriate surrogates for antitumor efficacy [169].

**Table 1:** Clinical trials utilizing innate immune agonists in combination with immunotherapies.

Agonist	Target	Combination	Indication	Delivery Route	Phase	NCT Code
SB11285	STING	Anti-PD-1	Advanced solid tumors	i.v.	Ia/Ib	NCT04096638 [156]
BI 1387446	STING	Anti-PD-1	Advanced, unresectable and/or metastatic malignant solid tumors	i.t.	I	NCT04147234 [170]
MK-1454 (Ulevostinag)	STING	Anti-PD-1	Advanced or metastatic solid tumors or lymphomas	i.t.	I	NCT03010176 [155]
MK-1454 (Ulevostinag)	STING	Anti-PD-1	Untreated metastatic or unresectable, recurrent HNSCC	i.t.	II	NCT04220866 [155]
SYNB1891	STING	Anti-PD-L1	Refractory advanced cancers	i.t.	I	NCT04167137 [171]
GSK3745417	STING	Anti-PD-1	Advanced solid tumor	N/A	I	NCT03843359
MK-2118	STING	Anti-PD-1	Refractory, advanced solid tumors or lymphomas	i.t. or s.c.	I	NCT03249792 [157]
IMSA101	STING	Anti-PD-L1	Advanced solid tumors	i.t.	I	NCT04020185 [172]
		Radiotherapy + anti-PD-1	Oligometastatic and oligoprogressive solid tumor malignancies	N/A	IIa	NCT05846646, NCT05846659 [173]
BMS-986301	STING	Anti-PD-1 Anti-CTLA-4	Advanced solid tumors	i.v., i.t., i.m.	I	NCT03956680
SNX281	STING	Anti-PD-1	Advanced solid tumors or lymphomas	i.v.	I	NCT04609579 [174]
TAK-676	STING	Anti-PD-1	Advanced or metastatic solid tumors	i.v.	I	NCT04420884 [175]
TAK-676	STING	Radiotherapy + anti-PD-1	Advanced NSCLC, TNBC, or HNSCC	i.v.	I	NCT04879849 [176]
	TLR2	CD19 CAR-T cells with intracellular signaling domains of CD28 and TLR2 (1928zT2 CAR-T cells)	Chemotherapy resistant or refractory CD19^+^ acute leukemia	i.v.	I	NCT02822326
	TLR2	CD19 CAR-T cells with intracellular signaling domains of CD28 and TLR2 (1928zT2 CAR-T cells)	Relapsed or refractory B-cell lymphoma	i.v.	I	NCT04049513 (ENABLE) [177]
Poly I:C	TLR3 RIG-I MDA-5	Anti-PD-1	Unresectable HCC	i.m.	II	NCT03732547 (CISLD-1)
BO-112	MDA-5	Anti-PD-1	Resectable soft tissue sarcoma	i.t.	I	NCT04420975 [178]
Rintatolimod	TLR3	Anti-PD-L1	Metastatic PDAC	i.v.	Ib/II	NCT05927142
G100	TLR4	Anti-PD-1 + IDO inhibitor +EZH2 inhibitor + metronomic cyclophosphamide	Advanced sarcoma	i.t.	II	NCT02406781 [179]
TransCon TLR7/8 agonist	TLR7/8	Anti-PD1	Advanced or metastatic solid tumors	i.t.	I/II	NCT04799054 [165]
Imiquimod	TLR7	Anti-PD-1	Stage IIIB-IV melanoma	Cutaneous	I	NCT03276832
Imiquimod	TLR7	Peptide vaccine	Advanced pancreatic cancer or colorectal	Topical	I	NCT02600949
BNT411	TLR7	Anti-PD-L1+ carboplatin + etoposide	Malignant solid tumors	i.v.	I/IIa	NCT04101357
SBT6050	TLR8	Anti-PD-1	Advanced HER2 expressing solid tumors	N/A	I/Ib	NCT04460456
MGN1703	TLR9	Anti-CTLA-4	Advanced solid tumors	s.c.	I	NCT0266877 [180]
SD-101	TLR9	Anti-PD-1 anti-CTLA-4 Anti-LAG-3	Locally advanced pancreatic adenocarcinoma, intrahepatic cholangiocarcinoma and HCC, uveal melanoma metastatic to the liver	Hepatic artery infusion using pressure enabled drug delivery	I/Ib	NCT05607953 NCT05220722 NCT04935229 [181,182,183]
CMP-001	TLR9	Anti-PD-1	Advanced or metastatic cancer, melanoma, metastatic castration resistant prostate cancer, advanced melanoma, recurrent or metastatic HNSCC, relapsed and refractory lymphoma	i.t., s.c.	I, II, III	NCT04916002 NCT04401995 NCT05445609 NCT04695977 NCT04698187 NCT04633278 NCT03983668 [166]
MK-4621	RIG-I	Anti-PD-1	Advanced solid tumors	i.t.	I	NCT03065023 NCT03739138 [168]

N/A: not available; i.v.: intravenous; i.t.: intratumoral; s.c.: subcutaneous; i.m.: intramuscular; HNSCC: head and neck squamous cell carcinoma; NSCLC: non-small cell lung cancer; TNBC: triple-negative breast cancer; HCC: hepatocellular carcinoma; PDAC: pancreatic ductal adenocarcinoma.

### 4.3. Augmenting Adoptive Cell Therapy with Innate Immune Modulation

Adoptive cell therapy (ACT), including CAR-T and NK cell therapies, has revolutionized treatment of hematologic malignancies yet efficacy in solid tumors remains largely disappointing. Barriers such as limited trafficking and infiltration, immune suppression within the TME, and physical obstacles, including dense extracellular matrix (ECM) and hypoxia, persist across studies, highlighting a recurring challenge that has not yet been overcome. Suppressive populations (Tregs, MDSCs, M2-like TAMs) and checkpoint ligands (PD-L1, VISTA, B7-H4) further compromise transferred cell function, raising questions about whether current strategies can meaningfully translate into durable clinical benefit [149,184].

#### 4.3.1. Innate Agonists as TME-Modulating Adjuvants

TLR and STING modulators have been evaluated as adjuvants to overcome TME-mediated resistance. For instance, the TLR3 agonist poly I:C improved CAR-T infiltration in breast and colon cancer models by attenuating MDSC-mediated suppression, yet the benefits were modest and dependent on intact IFN-I signaling [185]. Critically, durable antitumor control frequently required co-administration of PD-1 or MDSC-targeting antibodies with STING agonists, suggesting that these innate pathways alone are insufficient to overcome TME-mediated resistance [112]. Similarly, peri-tumoral or scaffold-based co-delivery of STING agonists with CAR-T cells promoted DC maturation, infiltration, and cytokine production, resulting in tumor regression in otherwise CAR-resistant models [112,186,187]. Nanovesicle- and hydrogel-based formulations extended this approach to orthotopic lung and breast cancers, enhancing CAR-T infiltration and reducing exhaustion [187,188].

Tumor-intrinsic STING activation has also been proposed to enhance ACT efficacy. In renal cell carcinoma models, DNA-damaging agents such as PARP inhibitors promoted cytosolic DNA release and cGAS–STING signaling, reportedly improving CAR-T efficacy [189]. In PDAC models, STING activation triggered mitochondrial apoptosis and sensitized tumor cells to CAR-NK mediated killing [190]. Similarly, the STING agonist IMSA101 induced IL-18 release from the TME, enhancing CAR-T cytotoxicity and cytokine production in murine melanoma [191] and PDAC, while in human tumor models, STING activation promoted NK cell recruitment via CXCR3-dependent chemotaxis, suggesting potential synergy with CAR-NK therapies [192]. Yet, the frequent epigenetic silencing of STING across human tumors casts doubt on the generalizability of these findings and underscores the need for pathway-based biomarkers in patient selection [159,160,161,162].

Activation of RIG-I in tumor cells triggers mitochondrial and extrinsic apoptosis, pyroptosis, and IFN-I signaling, sensitizing targets to CAR-T killing and promoting bystander antigen spread [193,194,195,196]. Most compellingly, Soliman et al. [196] demonstrated across diverse tumor models that loss of tumor-intrinsic RIG-I/MAVS is a major resistance mechanism to CAR-T cytotoxicity. Restoring this axis—via genetic means or exogenous agonists—enhanced CAR-T efficacy even under low antigen density or unfavorable effector-to-target ratios, suggesting that tumor-intrinsic RIG-I activity is deterministic rather than supportive for CAR-T function. By contrast, RIG-I activation within T cells themselves appears deleterious, limiting effector fitness and promoting apoptosis [197]. This dichotomy suggests that the therapeutic window for RIG-I agonists lies in stimulating tumor and myeloid compartments, not CAR-T cells directly.

To exploit this principle, Johnson et al. [198] engineered CAR-T cells to secrete EVs loaded with RN7SL1, a natural RIG-I/MDA5 ligand. This activated RIG-I in host DCs, depleted suppressive myeloid subsets, expanded endogenous tumor-reactive T cells, and controlled antigen-heterogeneous tumors. Synthetic ligands such as 3pRNA and SLR14 remodel the TME, recruit CD8^+^ and NK cells, and synergize with checkpoint blockade in preclinical models [199,200]. The mechanistic findings of Soliman et al. support these effects, showing that RIG-I/MAVS lowers the threshold for CAR-T engagement both through TME modulation and induction of tumor-intrinsic apoptotic pathways [196].

Pharmacologic RIG-I agonists enhance NK cytotoxicity via TRAIL and IFN induction [79,121]. Yet despite rapid clinical advances in CAR-NK platforms, no published studies have tested direct combinations with RIG-I agonism. Analogous to RN7SL1-armed CAR-T cells, NK-based carriers of RIG-I ligands could serve as targeted vectors, while systemic agonists might simultaneously prime NK function—a conceptually strong but untested strategy.

Early clinical testing of MK-4621/RGT100 confirmed target engagement and safety but demonstrated modest efficacy, likely due to systemic IFN-I driven toxicities [168]. Translation of direct RIG-I agonists therefore remains hampered by delivery and tolerability barriers. Similarly, while preclinical work supports the synergy of STING and TLR agonists with ACT, clinical translation remains limited by pathway silencing in human tumors, systemic toxicities, and the requirement for combination strategies to achieve durable responses.

#### 4.3.2. Engineering Innate Signaling into CAR Platforms

A parallel strategy is to embed innate signaling domains directly into CAR constructs. Incorporation of TLR2, MyD88/CD40, or related motifs enhances expansion, effector function, and persistence. For example, CAR-T cells with TLR2 domains showed improved adhesion, migration, and mTOR signaling, while those with MyD88/CD40 demonstrated potent responses in lymphoma models. The integration of a truncated TLR4 signaling costimulatory domain promotes CAR-T cell function against solid tumors [201]. To date, engineering CAR-T cells to incorporate signaling domains pertaining to RLR pathways has not been explored. Third-generation CAR-T cells with TLR2 domains are currently under clinical investigation (ENABLE trial) [202]. However, constitutive signaling raises the risk of uncontrolled activation, cytokine release syndrome (CRS), immune effector cell-associated neurotoxicity syndrome (ICANS), or premature exhaustion, highlighting the need for inducible or tunable circuits rather than permanent activation.

The same dualism applies to T cell intrinsic STING signaling. Endogenous cGAS–STING activity can promote CD8^+^ T cell stemness and improve anti-tumor responses [203], whereas in models using TCR transgenic or CAR-T cells, T cell intrinsic STING signaling facilitated Th1/Th9 differentiation and supported effector function [204]. However, chronic or high-dose activation of STING can induce calcium dysregulation, ER stress, and cell death [205]. For instance, DMXAA exposure at high concentrations (>5 µg/mL) triggered T cell apoptosis, and tumors have been shown to exploit STING-mediated T cell death as an immune evasion mechanism [206,207]. STING gain-of-function mutations (e.g., V155M) reveal antiproliferative effects in human T cells independent of IFN-I [208]. Interestingly, NK cells show resistance to STING-induced cytotoxicity due to rapid turnover of activated STING, making CAR-NK more compatible with STING agonist strategies [192]. While an intact STING pathway supports their persistence and function, excessive activation may impair viability and lead to exhaustion [209,210,211]. These contrasting effects underscore the need for precise temporal and spatial control of innate signaling in engineered products.

### 4.4. Tumor-Intrinsic Androgen Receptor Signaling Modulates Immune Evasion and Impacts Immunotherapy Outcomes

Tumor-intrinsic androgen receptor (AR) signaling functions as a profound modulator of immune evasion and resistance to immunotherapy, not merely by hormonal effects on cancer cell proliferation but through a complex reprogramming of tumor-intrinsic pathways that remodel the TME and suppress anti-tumor immunity. While the classical role of AR centers on hormone-driven cancers like prostate cancer, a growing body of evidence illuminates its oncogenic and immunomodulatory influence in traditionally hormone-independent cancers such as melanoma. This expanded role underscores a nuanced crosstalk between tumor-intrinsic signaling and immune evasion, critically shaping immunotherapy outcomes [212,213,214].

At the molecular level, AR signaling intricately governs immune evasion by orchestrating various tumor-intrinsic pathways. These pathways are not isolated phenomena but interwoven networks that collectively foster an immune suppressive milieu within the TME. Tumor-intrinsic oncogenic signaling pathways—encompassing AR, PI3K/AKT/mTOR, Wnt/β-catenin, and epigenetic modifiers—functionally converge to impede immune cell infiltration, suppress antigen presentation, and enhance the expression of immune checkpoint molecules, thus fostering an “immune cold” tumor phenotype resistant to immune-mediated eradication [215,216].

AR signaling directly influences the secretion and regulation of cytokines and chemokines, pivotal mediators of immune cell trafficking and activation. It skews the cytokine milieu toward an immunosuppressive landscape by elevating factors such as IL-6 and TNF-α while diminishing IFN-γ, which is essential for CTL function. This cytokine reprogramming dampens the recruitment and activation of effector immune populations including CD8^+^ T cells and NK cells, leading to decreased immune surveillance and increased tumor tolerance [214].

Moreover, AR signaling modulates key immune checkpoints at the tumor-cell level by upregulating molecules such as programmed death-ligand 1 (PD-L1) and B7-H3 [212]. These checkpoints serve as molecular shields that inhibit T cell activation and promote functional exhaustion, mechanisms that tumors exploit to evade immune destruction. This upregulation has been observed notably in castration-resistant prostate cancer and melanoma, resulting in diminished efficacy of ICI therapies.

A particularly compelling AR-mediated mechanism involves the evasion of NK cell cytotoxicity through modulation of ligand availability. AR activation induces the shedding of MICA/B, critical ligands recognized by the NKG2D receptor, via upregulation of metalloproteases like ADAM10. The shedding of these ligands from the tumor cell surface effectively cloaks them from NK cell-mediated killing, enabling the tumor to escape this key arm of innate immunity. Targeting AR or ADAM10 re-sensitizes tumors to NK cell attack and may restore immune control in resistant melanoma models [214].

Additionally, AR signaling interferes with the functional capacity of T cells themselves. It induces expression of USP18, a molecule that inhibits TAK1 kinase phosphorylation and downstream NF-κB activation, thereby weakening CD8^+^ T cell effector functions. Pharmacological inhibition of AR signaling has been shown to rescue these defects, restoring T cell cytotoxicity and enhancing synergy with anti-PD-1 therapies [217].

Beyond direct immunosuppressive signaling, AR influences the transcriptional and epigenetic landscape of the tumor to further generate immune-resistant states. Elevated AR activity represses genes related to antigen processing and presentation and interferon responses, effectively putting the tumor into an immune-deserted state that resists infiltration by effector T cells. This process also overlaps with resistance to targeted therapies like BRAF and MEK inhibitors in melanoma, highlighting that tumor-intrinsic pathways shaped by AR contribute broadly to therapeutic resistance [212,218].

These tumor-intrinsic signaling pathways do not act in isolation; rather, they combine with other oncogenic and epigenetic alterations to sculpt an immune microenvironment hostile to immune attack. For instance, the loss of tumor suppressors such as PTEN, common in diverse cancers, activates PI3K/AKT/mTOR pathways that enhance PD-L1 expression and inhibit T cell infiltration, working in concert with AR signaling to deepen immunosuppression. Meanwhile, β-catenin activation restricts dendritic cell recruitment, further preventing initiation of effective anti-tumor immunity. Consequently, tumors leverage a network of intrinsic pathways to evade immune recognition and resist immunotherapy through mechanisms including immune exclusion, impaired antigen presentation, and functional immune suppression [214,215].

Clinically, these molecular insights have profound implications. The negative association between high AR activity and immune infiltration correlates with poor responses to immune checkpoint blockade across cancer types. Males, bearing higher systemic androgen levels, frequently exhibit lower response rates to ICI, further implicating AR biology in observed sex-based disparities in immunotherapy efficacy. Moreover, serum biomarkers reflecting AR-driven processes—such as elevated soluble MICA—have been linked to poor outcomes in melanoma patients receiving PD-1 inhibitors like pembrolizumab [212,219].

Emerging clinical trials (NCT04191096, NCT04471974) are now exploring combination strategies that integrate AR inhibition with immunotherapy to overcome these resistance mechanisms. For example, combining AR antagonists such as enzalutamide or apalutamide with PD-1 or PD-L1 inhibitors is under evaluation in prostate cancer and is extending into melanoma cohorts [220,221]. Preclinical models demonstrate that androgen deprivation therapy (ADT) can remodel the TME, increase immune cell infiltration, and enhance the efficacy of checkpoint blockade, supporting these clinical initiatives. Additionally, approaches targeting metalloproteases responsible for MICA shedding offer another therapeutic avenue to restore NK cell-mediated cytotoxicity in AR-high tumors [128,222].

### 4.5. Integrative Perspectives on Overcoming Clinical Barriers Through Combination Innate Immunotherapy

Harnessing innate immunity to potentiate cancer immunotherapy represents a promising strategy to overcome resistance mechanisms and improve patient outcomes, but clinical translation remains fraught with hurdles. Delivery barriers are among the most formidable. Many innate agonists, such as STING and TLR agonists, still rely heavily on i.t. administration. While this route allows localized immune activation and reduces systemic toxicities like CRS, it is impractical for patients with visceral or metastatic lesions. Systemic formulations have struggled to achieve sufficient drug exposure without unacceptable toxicities, including cardiovascular complications and autoimmunity. Although NP encapsulation, biomaterial scaffolds, OVs, and cell-based vectors hold promise for improving systemic delivery, these approaches remain largely preclinical.

Even when delivered effectively, tumor-intrinsic barriers often undermine efficacy. Frequent epigenetic silencing of innate pathways such as STING restricts the therapeutic benefit largely to non-malignant stromal or immune compartments, leading to heterogeneous patient responses. Similarly, overstimulation of TLR pathways risks triggering counter-regulatory immunosuppressive circuits—including MDSC expansion and checkpoint ligand upregulation—that blunt long-term efficacy. RIG-I agonists face an even wider translational gap, with encouraging preclinical activity failing thus far to yield clinical responses. Collectively, these experiences highlight recurring barriers that transcend individual pathways: delivery challenges, tumor-intrinsic silencing, counter-regulatory feedback, and the inadequacy of preclinical models to fully capture human heterogeneity.

The limitations of current biomarker strategies further compound these issues. Pharmacodynamic readouts such as IFN-I signatures, ISG induction, or transient cytokine surges (e.g., IFN-γ, CXCL10, IL-6) consistently confirm target engagement but have failed to correlate reliably with durable clinical benefit. This disconnect raises fundamental concerns about whether current biomarkers adequately capture the regulatory feedback loops that shape therapeutic outcome. As a result, patient selection in many trials has been essentially random, diluting observable efficacy and obscuring true responder populations. Precision biomarker development to detect intact innate signaling, identify tumor-intrinsic vulnerabilities, and stratify patients remains an urgent unmet need.

Because innate immune signaling can promote either tumor clearance or immune evasion depending on its magnitude and duration, carefully calibrated dosing regimens and scheduling are essential to prevent paradoxical inflammation or immune exhaustion. Moreover, tumor-intrinsic signaling networks—such as the NKG2D ligand axis or androgen receptor pathways—constitute underexplored but important modulators of immune resistance that may guide rational combination strategies. Indeed, durable efficacy is most likely to emerge when innate agonists are integrated into combinatorial regimens with checkpoint inhibitors, adoptive cell therapies, chemotherapy, radiotherapy, or metabolic modulators.

Taken together, the clinical experiences with STING, TLR, and RIG-I agonists underscore the need for a more rigorous, comparative translational framework linking mechanistic context to patient outcome. Proof-of-mechanism—such as cytokine induction or transient immune infiltration—has been repeatedly demonstrated, but proof-of-concept efficacy in patients remains elusive. Without advances in systemic delivery, refined biomarker-guided patient selection, and a deeper mechanistic understanding of pathway-specific feedback, these agents risk remaining immunological adjuvants rather than durable therapeutics.

## 5. Conclusions

Innate immune reprogramming remains one of the most compelling avenues to expand the reach of cancer immunotherapy. The last decade has clearly established proof of mechanism, but translating this into durable patient benefit requires moving beyond isolated pathway activation toward a more integrated framework. Delivery innovations, while necessary, will not be sufficient unless coupled with precise patient stratification and a deeper understanding of how oncogenic signaling circuits intersect with innate sensing. Future progress will hinge on embedding innate agonists into rational combinations and next-generation cellular therapies, where their ability to mobilize dendritic and cytotoxic programs can be amplified and sustained. Equally important is the shift from descriptive biomarkers to predictive ones that can align the mechanistic context with the clinical outcome. Such advances will enable the field to progress from demonstrating transient immune activation to achieving durable disease control in heterogeneous patient populations.

At present, however, critical uncertainties remain: the challenge of distinguishing tumor-intrinsic from stromal or myeloid contributions, the recurrent silencing of key pathways such as STING, and the absence of biomarkers that reliably identify patients most likely to respond. Overcoming these barriers will require more than technical refinements—it will demand a reorientation of the field toward biomarker-guided strategies that integrate genomic and functional insights, coupled with delivery platforms capable of achieving systemic exposure without compromising safety. Just as importantly, future research must unravel how innate sensing intersects with oncogenic and metabolic circuits, since these interactions dictate whether inflammation drives tumor clearance or immune escape. By embedding these mechanistic insights into carefully designed combinations—not only with checkpoint blockade but also with metabolic, stromal, and epigenetic modulators—innate agonists can be transformed from immunological adjuvants into durable therapeutic cornerstones.

The field now stands at an inflection point: continued incremental progress will yield only transient immune activation, whereas a concerted effort to integrate delivery, biomarkers, and mechanistic context could redefine innate immune modulation as a foundational pillar of immunotherapy. Meeting this challenge offers the opportunity not merely to broaden the reach of current therapies but to reshape the landscape of resistance itself, expanding the fraction of patients who can achieve long-term benefit.

## Data Availability

No new data were created or analyzed in this study. Data sharing is not applicable to this article.
